# PRDX2 Protects Against Atherosclerosis by Regulating the Phenotype and Function of the Vascular Smooth Muscle Cell

**DOI:** 10.3389/fcvm.2021.624796

**Published:** 2021-03-11

**Authors:** Jing Li, Cong Wang, Wenjing Wang, Lingzi Liu, Qingqing Zhang, Jun Zhang, Bo Wang, Shujing Wang, Li Hou, Chuanzhou Gao, Xiao Yu, Lei Sun

**Affiliations:** ^1^Department of Pathology and Forensic Medicine, College of Basic Medical Sciences, Dalian Medical University, Dalian, China; ^2^Department of Biochemistry and Molecular Biology, College of Basic Medical Sciences, Institute of Glycobiology, Dalian Medical University, Dalian, China; ^3^Department of Electron Microscope, Dalian Medical University, Dalian, China

**Keywords:** peroxiredoxin 2, atherosclerosis, vascular smooth muscle cell, phenotype, MAPK signaling, ROS

## Abstract

Peroxiredoxin 2 (PRDX2), an inhibitor of reactive oxygen species (ROS), is potentially involved in the progression of atherosclerosis (AS). The aim of this study was to explore the role and mechanism of PRDX2 in AS. The expression of PRDX2 was evaluated in 14 human carotid artery tissues with or without AS. The results showed that the positive reaction of PRDX2 was observed in the carotid artery vascular smooth muscle cells (CAVSMCs). To assess the mechanism by which PRDX2 may function in AS, the CAVSMCs were transfected with pEX4-PRDX2 and si-PRDX2. The catalase, hydrogen peroxide (H_2_O_2_) scavenger, was used to further confirm that PRDX2-induced inhibitory effects might be mediated through reducing ROS levels. Phenotype alteration and functional testing included transcription testing, immunostaining, and expression studies. The drug of MAPK signaling pathway inhibitors SB203580, SP600125, and PD98059 was used to evaluate the underlying mechanism. In this study, we found that the protein level of PRDX2 and the level of H_2_O_2_ were higher in the human AS carotid artery tissues than in the normal carotid artery tissues, accompanied with the activation of MAPK signaling pathway. The up-regulation of PRDX2 in the CAVSMCs significantly decreased the expression of ROS, collagen type I (COL I), collagen type III (COL III), vascular cell adhesion molecule-1 (VCAM-1), and intercellular adhesion molecule-1 (ICAM-1) and inhibited the proliferation, migration, and transformation of the CAVSMCs. The up-regulation of PRDX2 reversed the effect of the CAVSMCs treated with tumor necrosis factor-α (TNF-α). In addition, PRDX2 down-regulation promoted the protein levels of p-p38, p-JNK, and p-ERK, which was confirmed in relevant MAPK inhibitor treatment experiments. Our results suggest a protective role of PRDX2, as a scavenger of ROS, in AS progression through inhibiting the VSMC phenotype alteration and function *via* MAPK signaling pathway.

## Introduction

Peroxiredoxin (PRDX) is an antioxidant protease containing important catalytic cysteine residues, which can be used to remove hydrogen peroxide (H_2_O_2_), lipid H_2_O_2_, and peroxynitrite (1, 2). There are six subtypes of the PRDX family in mammals, which are generally divided into 1-cys and 2-cys according to the number of cysteine residues, among which PRDX 1–5 belong to 2-cys subtype, and PRDX 6 belongs to 1-cys subtype (3). PRDX2, as one of the most efficient reactive oxygen species (ROS) scavengers among all PRDXs, is involved in many pathological processes (4, 5). ROS are composed of superoxide anion and H_2_O_2_ and participate in the occurrence and development of atherosclerosis (AS) by impairing cellular functions (6, 7). PRDX2 has been identified as having antioxidant properties in various organisms (1, 6, 8). PRDX2 is susceptible to excessive oxidation of the cells, which may lead to accelerated AS due to the failure to eliminate ROS (9). In addition, PRDX2 expression was more pronounced in the endothelial cells and immune cells in atheroid-prone areas and plaques (10). However, it remains unclear the mechanism underlying the role of PRDX2 in the development of AS.

AS is characterized by lipid deposition in the intima; the formation of plaques; the deposition of collagen; and the accumulation of lipids, cholesterol, foamy cells, and so on. Dominant in vascular wall collagens are the main types of fiber formation, namely, collagen type I (COL I) and collagen type III (COL III) (11). COL III is thought to be associated with the ductility of blood vessel walls, whereas COL I may cause hardening of the arteries. It has been reported that the vascular smooth muscle cell (VSMC) in AS lesions produced a new extracellular matrix (ECM) that is essentially collagen (12). Vascular cell adhesion molecule-1 (VCAM-1) and intercellular adhesion molecule-1 (ICAM-1) promoted the development of AS, enhancing with the aggravation of lesions. The role of PRDX2 in AS, as it relates to VSMCs, COL I, COL III, VCAM-1, and ICAM-1, remains largely unknown.

Here, we evaluated the expression and roles of PRDX2 in the human carotid artery. We applied exogenous PRDX2 to the carotid artery vascular smooth muscle cells (CAVSMCs) to analyze the effects of PRDX2 on cell proliferation, migration, phenotype shift, and functional alteration. Subsequently, to explore the possible molecular mechanism involved in the progression of AS, we applied tumor necrosis factor-α (TNF-α)-induced medium to the CAVSMCs and analyzed the relative levels of ROS, collagen synthesis, VCAM-1, ICAM-1, and MAPK pathway molecules.

## Materials and Methods

### Tissue and Cell Culture

Control samples (*n* = 7, 6 males and 1 female, ages 27–52 years), the phase of AS experimental samples (*n* = 7, 7 males, ages 30–73 years), and their carotid artery tissue were collected within 48 h after death by the Department of Pathology and Forensic Medicine, Dalian Medical University, between 2017 and 2020 (Supplementary Figure 1A, Supplementary Material 1). All procedures that involved human samples conformed to the principles outlined in the Declaration of Helsinki. The study was approved by the Ethics Committee of Dalian Medical University. Human CAVSMCs derived from the carotid artery of healthy adults were purchased from Bioleaf (Shanghai, China). The cells were cultured in 15% Dulbecco's modified Eagle's medium (DMEM; Gibco, USA) containing 15% fetal bovine serum (FBS; Gibco, USA) in a 37°C incubator and 5% CO_2_. The experiment cells were passaged 1–20 times. TNF-α (Proteintech, China), catalase, SB203580, SP600125, PD98059 (MCE, China), and other transfection reagents (GenePharma) were used to treat the cells according to the experimental design.

### Histological Analysis

The human carotid artery tissues were fixed in formalin, embedded in paraffin, and cut cross-sectionally into 3.0–4.0 μm thick sections. Histological examination was performed with Hematoxylin and Eosin (H&E), Elastic Van Gieson (EVG), and Masson's trichrome staining. For immunohistochemistry (IHC) analysis, the tissues were incubated overnight at 4°C with antibodies against PRDX2 (1:100; Abcam, USA), OPN (1:100; Proteintech, China), and smooth muscle protein 22 alpha (SM22α) (1:200; Proteintech, China).

### Measurement of ROS

#### H_2_O_2_ Assay

The level of H_2_O_2_ was measured by chemical colorimetry using a Hydrogen Peroxide Assay Kit (Beyotime Institute of Biotechnology, Shanghai, China, S0038). According to the manufacturer's protocol, 100 μl of supernatant was added into a 96-well plate and incubated with 100 μl of reaction solution at room temperature for 30 min, and the absorbance of 560 nm was measured. The concentration of H_2_O_2_ was obtained from the standard concentration curve with three independent experiments.

#### Fluorometric Intracellular ROS

The cells were seeded into 24-well plates at a density of 5.0 ×10^4^ cells/well and incubated overnight. The level of intracellular ROS was measured by the Fluorometric Intracellular ROS Kit (MAK-143; Sigma, USA) according to the manufacturer's instructions. In brief, add 500 μl/well (10 μmol/L) of Master Reaction Mix into a cell plate and incubate for 20 min in a 37°C incubator and 5% CO_2_.

### Cell Transfection

PRDX2 full-length (pEX4-PRDX2) plasmid, PRDX2 small interfering RNA (siRNA-PRDX2-homo-749), and a non-targeting sequence (negative control, NC) were synthesized by GenePharma (China). The siRNA sequences shown in Table 1 were inoculated into 6-well plates (Corning, China) until their growth densities ranged from 50 to 70% and then transfected with Lipofectamine 2,000 reagent (Invitrogen, Life Technologies, NY, USA) and siRNA or plasmid as shown. After incubation for 6 h, DMEM/HIGH GLUCOSE (HyClone, Logan, UT, USA) medium containing 10% FBS (Gibco, USA) was used.

**Table 1:** Sequences of transfection.

**Sequence**		**5′-3′**
si-PRDX2	Sense	5′-GCAAGGAAUAUUUCUCCAATT-3′
	Antisense	5′-UUGGAGAAAUAUUCCUUGCTT-3′
si-NC	Sense	5′-UUCUCCGAACGUGUCACGUTT-3′
	Antisense	5′-ACGUGACACGUUCGGAGAATT-3′

### qRT-PCR

Total RNA was extracted from the human carotid artery tissue, and the CAVSMCs were cultured *in vitro* with TriZol reagent (Thermo Scientific). The concentration and quality of RNA were measured according to the NanoDrop spectrophotometer (ND-100; Thermo Scientific). cDNA was synthesized by total RNA. The PCR primer sequences are listed in Table 2. The expression of mRNA was quantified by using real-time quantitative reverse transcription PCR (qRT-PCR) using TransStart Top Green qPCR SuperMix (Trans).

**Table 2:** Primer sequences used in the study.

**Primer**	**Sense**	**Sequence 5′-3′**
PRDX2	F	5′-CACCTGGCTTGGATCAACACC-3′
	R	5′-CAGCACGCCGTAATCCTCAG-3′
GAPDH	F	5′-GTGGAAGGACTCATGACCACAGT-3′
	R	5′-GGAAGGCCATGCCAGTGA-3′

### Western Blot Analysis

The cells are cleaved, and proteins were extracted from the supernatant. The protein was quantified by a BCA Protein Assay Kit (Beyotime). Then, 50 μg of total cell protein dissolution products was isolated using sodium dodecyl sulfate-polyacrylamide gel electrophoresis (SDS-PAGE) and transferred onto nitrocellulose (NC) membranes (PALL). Glyceraldehyde-3-phosphate dehydrogenase (GAPDH) antibody was diluted at 1:4,000; PRDX2 (1:1,000; Abcam, USA and Proteintech, China), COL I (1:1,000; Abcam, USA), COL III (1:1,000; Proteintech, China), VCAM-1 (1:1,000; Abcam, USA), ICAM-1 (1:1,000; Abcam, USA), p-p38 (1:1,000; Abcam, USA), p38 (1:1,000; Abcam, USA), p-JNK (1:1,000; Abcam, USA), JNK (1:1,000; Abcam, USA), p-ERK (1:2,000; Proteintech, China), and ERK (1:1,000; Proteintech, China) antibodies were incubated at 4°C overnight after washing three times with Tris-buffered saline–Tween-20 (TBST). The membranes were incubated with the corresponding horseradish peroxidase (HRP)-conjugated secondary antibody (1:5,000; GE, HyClone) at 37°C for 1 h. The protein bands were visualized by enhanced chemiluminescence (ECL, Advansta) using a ChemiDoc™ MP imaging system (BIO-RAD).

### Cell Proliferation Assay

The CAVSMCs (1.0 ×10^5^/well) were seeded into a 96-well plate. According to the experimental proposal, cell proliferation was assessed using a Cell Counting Kit-8 (CCK-8; Trans). Then, 10 μl of CCK-8 solution was augmented to each well and incubated for 2 h in a humidified incubator. Cell proliferation assays were analyzed using a microplate reader (Thermo Scientific).

### Cell Migration Assay

The cell migration used a transwell chamber with a polycarbonic membrane (6.5 mm diameter and 8 μm pore size; Corning). The serum-free DMEM of no-transfected or transfected cell was added to the upper chamber, and 600 μl of 15% FBS–DMEM was added to the lower chamber. The cells that were migrated to the lower chamber surface were fixed with 4% methanol and stained with 10% Giemsa (Solarbio, China), and the unmigrated cells on the membrane were wiped off with cotton swabs.

### Immunofluorescence Staining

Immunofluorescence staining was analyzed using an Olympus microscope. The expression and distribution of PRDX2 (1:100; Abcam, USA and Proteintech, China), SM22α (1:100; Proteintech, China), and α-smooth muscle actin (α-SMA, 1:200; Proteintech, China) were detected. Moreover, the osteopontin (OPN, 1:100; Abcam, USA) was evaluated to mark the phenotype switching of the CAVSMCs. The nuclei were stained with 0.5 μg/ml 4′,6-diamidino-2-phenylindole (DAPI; Solarbio, China).

### Statistical Analysis

Data, shown as mean ± standard error of the mean, were analyzed with *t*-test or analysis of variance (ANOVA) to determine the significance of differences. Inter-group comparisons were performed using a one-way ANOVA for results from at least three independent experiments. Differences were considered statistically significant at a *p*-value of <0.05. All data were performed using GraphPad Prism 8.0.

## Results

### Human Carotid Artery Samples Have Higher PRDX2 Than Normal Artery Samples and Alteration of the VSMC in AS

**Figure 1:**
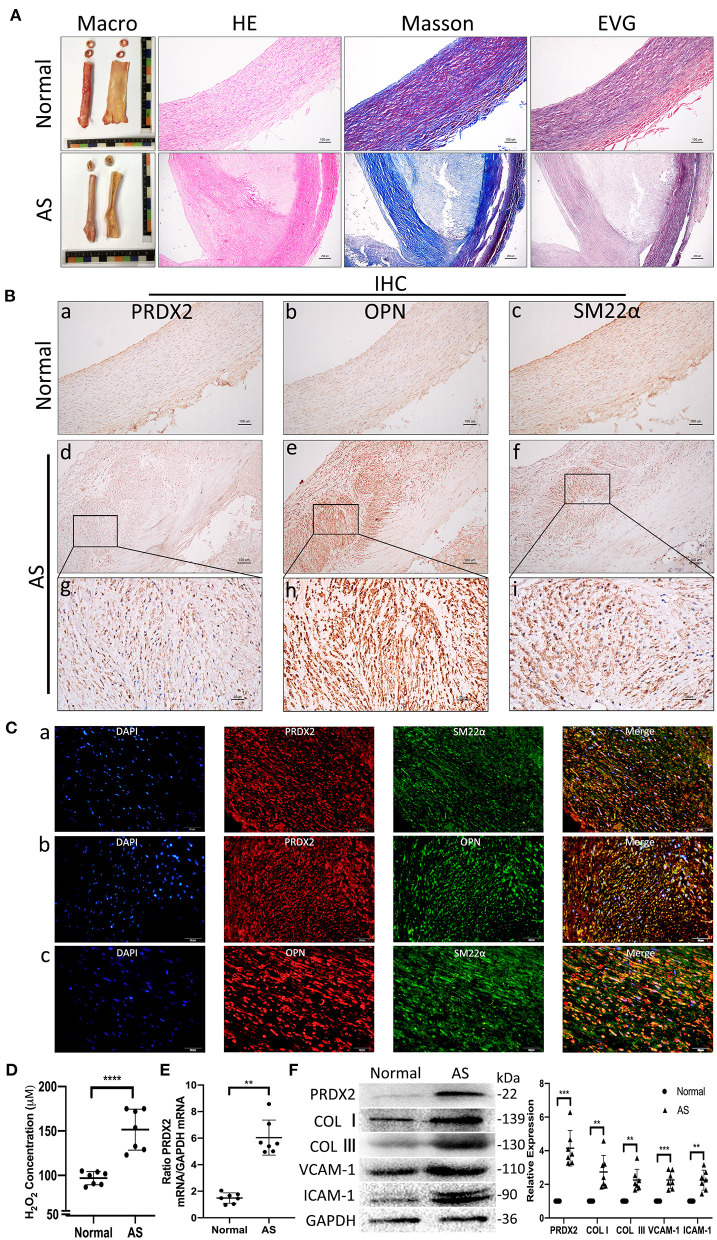
Human carotid artery samples have higher PRDX2 than normal artery samples and alteration of the VSMC in AS. **(A)** Human carotid artery tissue samples. Representative images of H&E, EVG, and Masson's trichrome staining. In Masson's trichrome staining, collagen fibers were stained blue, and myocardium was stained red. Scale bar: 100 and 250 μm. **(B)** Representative images of IHC staining of PRDX2, OPN, and SM22α. (a–f) Scale bar: 100 μm. (g–i) Scale bar: 25 μm. **(C)** Representative confocal immunofluorescence pictures. (a) PRDX2 (red) and SM22α (green). (b) PRDX2 (red) and OPN (green). (c) OPN (red) and SM22α (green). Nuclei were stained with DAPI (blue). Scale bar: 20 μm. **(D)** The concentration of H_2_O_2_ was evaluated by a Hydrogen Peroxide Assay Kit. **(E)** PRDX2 expression was assessed by qRT-PCR. **(F)** Protein expression levels of PRDX2, COL I, COL III, VCAM-1, and ICAM-1 were determined by Western blot. The data were obtained from three independent experiments (*n* = 7 humans per group, ^**^p < 0.01, ^***^p < 0.001, and ^****^p < 0.0001 vs. normal group).

According to H&E, EVG, and Masson staining, the expression of collagen was markedly increased in both the intima and the media of the human AS tissue compared with the human normal artery tissue (Figure 1A). IHC staining illustrated that the VSMCs expressed high levels of PRDX2 and OPN in the human AS tissue (Figure 1B). The intima VSMCs showed organ-like arrangement proliferation with higher expression of PRDX2, OPN, and SM22α. Consistent with IHC data, Figure 1C showed that PRDX2 and OPN expression was higher in the VSMCs of AS tissue than in the normal tissue (Supplementary Figures 1B,C). In order to evaluate the levels of ROS in AS tissue, the H_2_O_2_ concentration was determined. The results showed that the H_2_O_2_ concentration was increased in the human AS tissue compared with the human normal artery tissue (Figure 1D). qRT-PCR analysis showed that PRDX2 expression was significantly higher in the human AS tissue than in the human normal artery tissue (Figure 1E). The protein expression levels of PRDX2, COL I, COL III, VCAM-1, and ICAM-1 were markedly increased in the human AS tissue compared with the human normal artery tissue (Figure 1F). Collectively, these results suggest that PRDX2 may play potential protective roles in the progression of AS, which is related to the increasing of ROS and phenotype alteration of the VSMCs.

### PRDX2, as ROS Scavenger, Is Involved in the Process of TNF-α-Induced CAVSMCs Proliferation, Migration, Transformation, and Collagens Excessive Accumulation

**Figure 2:**
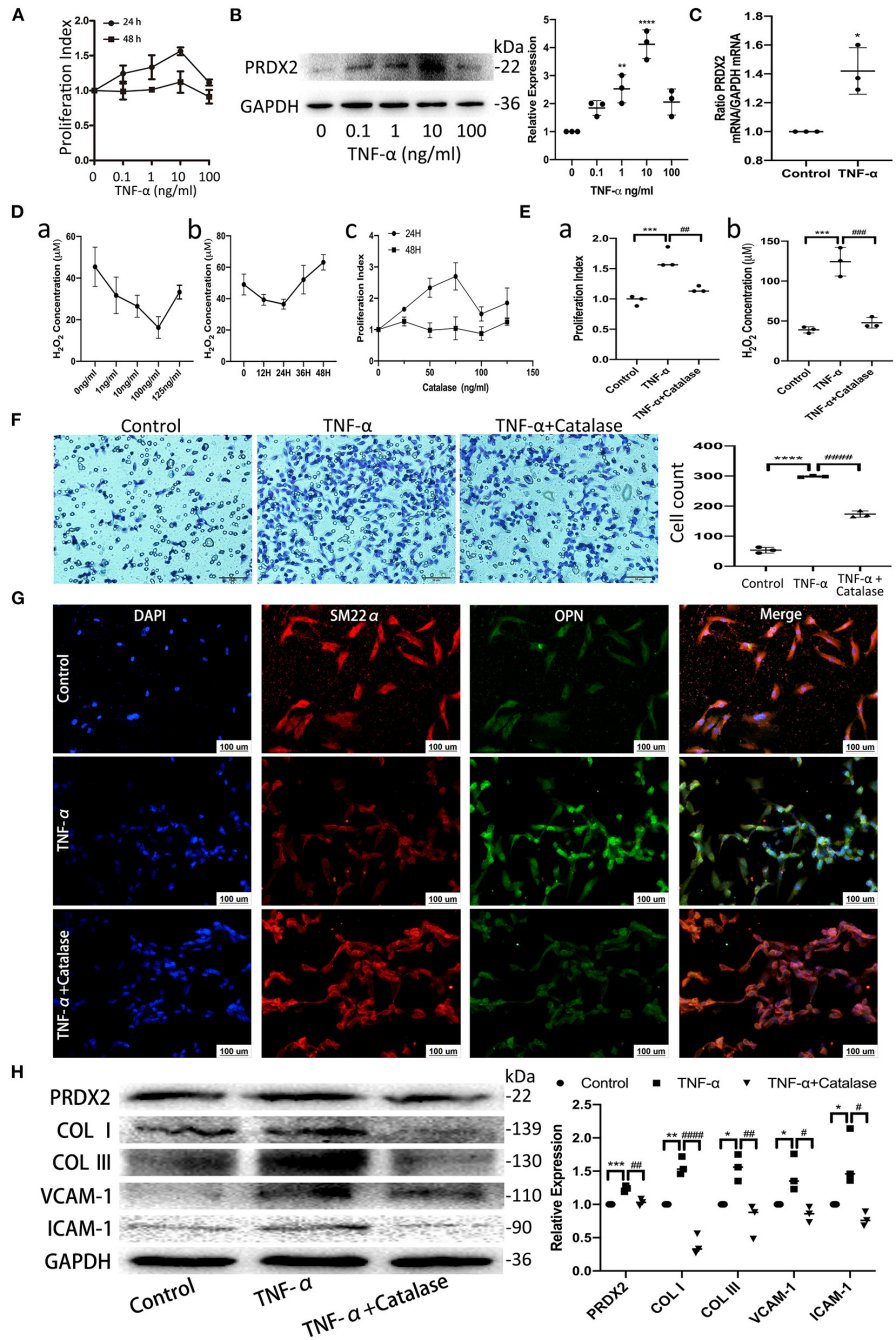
PRDX2, as ROS scavenger, is involved in the process of TNF-α-induced CAVSMCs proliferation, migration, transformation, and collagens excessive accumulation. **(A)** Effects of different concentrations of TNF-α on the proliferation of the CAVSMCs. **(B)** PRDX2 protein expression in response to different concentrations of TNF-α (^**^p < 0.01 and ^****^p < 0.0001 vs. control group). **(C)** PRDX2 expression was assessed by qRT-PCR (^*^p < 0.05 vs. control group). **(D)** (a) Effects of different concentrations of catalase on the production of H_2_O_2_ of the CAVSMCs. (b) Effects of different times of catalase (100 ng/ml) on the level of H_2_O_2_ of the CAVSMCs. (c) Effects of different concentrations of catalase on the proliferation of the CAVSMCs. **(E)** The reversion of catalase in the proliferation and the level of H_2_O_2_ of the CAVSMCs after TNF-α treatment (^***^p < 0.001 vs. control group, ^##^*p* < 0.01 and ^###^*p* < 0.001 vs. TNF-α treatment group). **(F)** Transwell migration assay of the CAVSMCs treated with TNF-α and catalase (^****^p < 0.0001 vs. control group, ^####^*p* < 0.0001 vs. TNF-α treatment group). **(G)** Representative confocal immunofluorescence pictures of SM22α (red) and OPN (green). Nuclei were stained with DAPI (blue). Scale bar: 100 μm. **(H)** Protein expression of PRDX2, COL I, COL III, VCAM-1, and ICAM-1 was determined by Western blot (^*^p < 0.05, ^**^p < 0.01, and ^***^p < 0.001 vs. control group, ^#^*p* < 0.05, ^##^*p* < 0.01, and ^####^*p* < 0.0001 vs. TNF-α treatment group). The data were obtained from three independent experiments.

TNF-α promotes apoptosis, proliferation, and collagen synthesis and secretion, ultimately leading to AS (13). Robust CAVSMCs proliferation was achieved when the cells were treated with TNF-α (10 ng/ml, 24 h) (Figure 2A). PRDX2 expression was analyzed at various concentrations of TNF-α after 24 h of treatment. As shown in Figure 2B, 10 ng/ml TNF-α significantly increased PRDX2 protein expression. qRT-PCR analysis showed that PRDX2 expression increased in the CAVSMCs treated with TNF-α concentration of 10 ng/ml after 24 h (Figure 2C). To further confirm that PRDX2-induced inhibitory effects might be mediated through reducing ROS levels, the catalase was used to inhibit H_2_O_2_. Catalase at a concentration of 100 ng/ml significantly decreased the H_2_O_2_ concentration and proliferation of the CAVSMCs after 24 h (Figure 2D). Proliferation, H_2_O_2_ concentration, and migration were decreased in the CAVSMCs treated with TNF-α and catalase compared with those treated with TNF-α after 24 h (Figures 2E,F). The transformation in TNF-α-induced CAVSMCs was shown by immunofluorescence assay. The higher expression of OPN was observed in TNF-α treatment, whereas the lower expression of OPN was observed in TNF-α and catalase treatments (Figure 2G). Furthermore, the Western blot assays indicated that the expression of PRDX2, COL I, COL III, VCAM-1, and ICAM-1 was significantly higher in the TNF-α-treated group than in the control group, which was reversed by catalase treatment (Figure 2H). These demonstrated that the inhibition of ROS was able to reverse the effects of TNF-α treatment. Taken together, these results indicate that PRDX2, ROS scavenger, might be involved in the TNF-α-induced progression of AS.

### Successfully Regulate PRDX2 in the CAVSMCs

**Figure 3:**
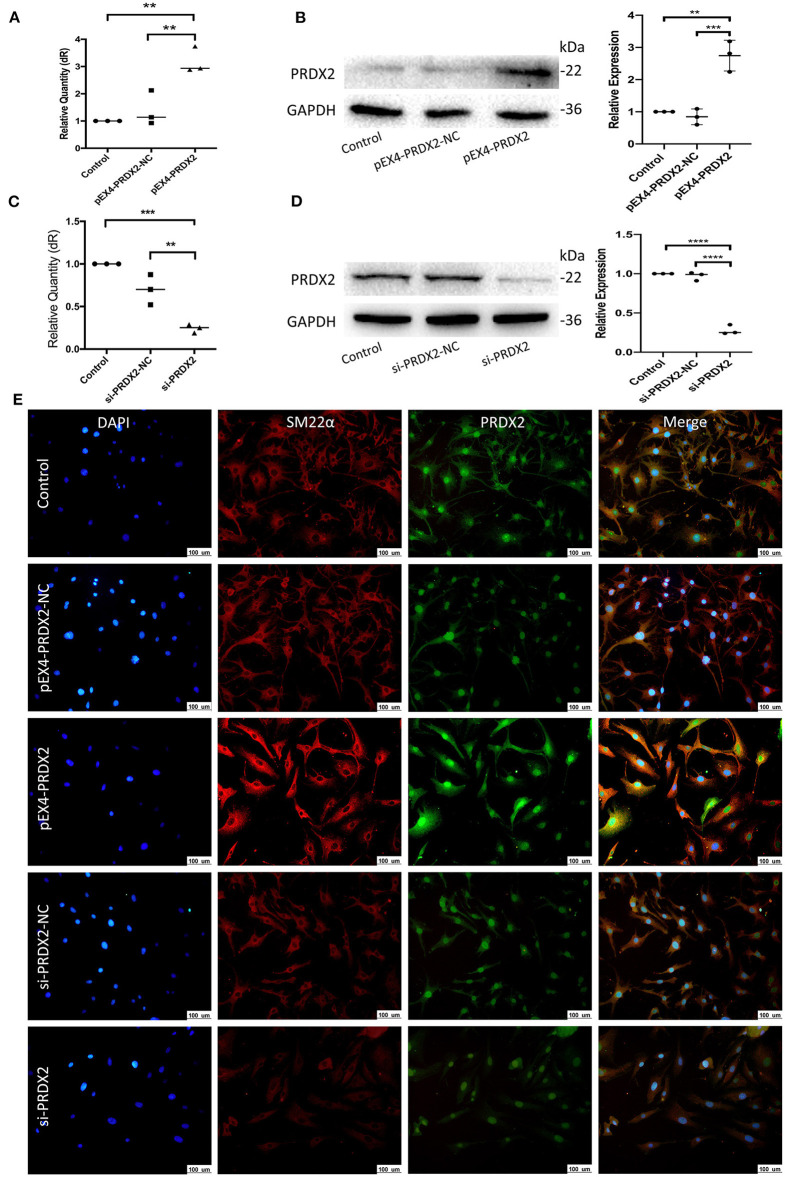
Successfully regulate PRDX2 in the CAVSMCs. **(A)** PRDX2 expression was assessed by qRT-PCR. (^**^p < 0.01 vs. control group and NC group). **(B)** Expression levels of PRDX2 protein in different groups according to Western blot (^**^p < 0.01 vs. control group and ^***^p < 0.001 vs. NC group). **(C)** PRDX2 expression were assessed by qRT-PCR (^***^p < 0.001 vs. control group and ^**^p < 0.01 vs. NC group). **(D)** PRDX2 protein expression in different groups was determined by Western blot (^****^p < 0.01 vs. control group and NC group). **(E)** Representative confocal immunofluorescence pictures of SM22α (red) and PRDX2 (green). Nuclei were stained with DAPI (blue). Scale bar: 100 μm.

To further explore the role of PRDX2 in AS, PRDX2 was over-expressed with pEX4-PRDX2 and knocked down with si-PRDX2 (siRNA-PRDX2-homo-729) transfection in the CAVSMCs. The transfection efficiency of PRDX2 was demonstrated using qRT-PCR and Western blot assays. The results indicated that the expression of PRDX2 was successfully over-expressed in the pEX4-PRDX2 and silenced in the si-PRDX2 compared with that in the control group and the respective NC group (Figures 3A–D). As shown in Figure 3E, the expression of PRDX2 in the CAVSMCs was consistent with qRT-PCR and Western blot. Additionally, the immunofluorescence staining revealed that SM22α was up-regulated in pEX4-PRDX2-CAVSMCs and down-regulated in si-PRDX2-CAVSMCs (Figure 3E). This result indicates that PRDX2 is involved in the transformation of the CAVSMCs.

### PRDX2 Attenuates ROS and Potentially Protects Against the Development of AS

**Figure 4:**
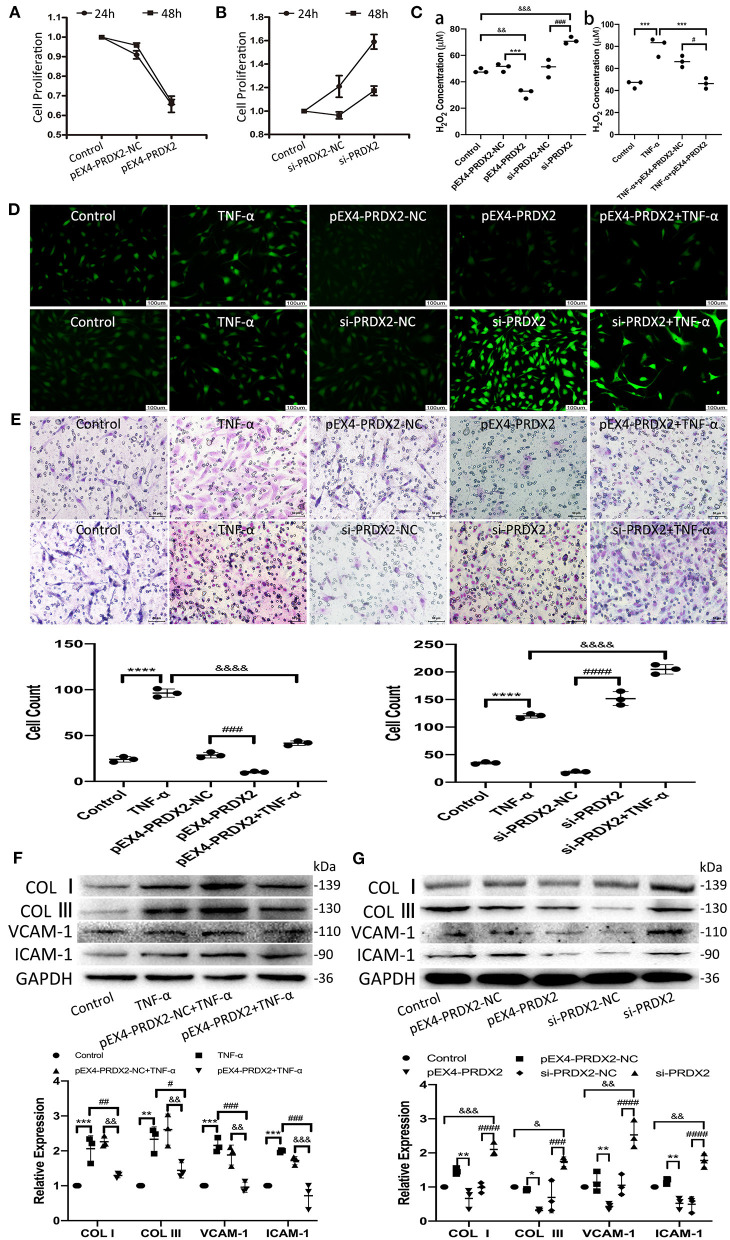
PRDX2 attenuates ROS and potentially protects against the development of AS. **(A,B)** CCK-8 assay was applied to evaluate endogenously expressed PRDX2 on the proliferation of the CAVSMCs. **(C)** The H_2_O_2_ concentration on pEX4-PRDX2 and si-PRDX2 transfection CAVSMCs and TNF-α treatment to evaluate the effect of PRDX2 in inhibiting ROS [(a) ^&&^*p* < 0.01 and ^&&&^*p* < 0.001 vs. control group, ^***^p < 0.001 vs. pEX4-PRDX2-NC group, ^###^*p* < 0.001 vs. si-PRDX2-NC group. (b) ^***^p < 0.001 vs. TNF-α group and ^#^*p* < 0.05 vs. TNF-α+pEX4-PRDX2-NC group]. **(D)** Representative immunofluorescence pictures of ROS (green). Scale bar: 100 μm. **(E)** Transwell assay was used on TNF-α treatment, pEX4-PRDX2, and si-PRDX2 transfection CAVSMCs to evaluate the migration effect of PRDX2 (^****^p < 0.0001 vs. control group, ^###^*p* < 0.001 and ^####^*p* < 0.0001 vs. respective NC group, ^&&&&^*p* < 0.0001 vs. TNF-α group). **(F)** Western blot and semi-quantitative analyses for COL I, COL III, VCAM-1, and ICAM-1 expression in pEX4-PRDX2-CAVSMCs treated with TNF-α (^**^p < 0.01 and ^***^p < 0.001 vs. control group, ^#^*p* < 0.05, ^##^*p* < 0.01, and ^###^*p* < 0.001 vs. TNF-α group, ^&&^*p* < 0.01 and ^&&&^*p* < 0.001 vs. pEX4-PRDX2-NC+TNF-α group). **(G)** Western blot and semi-quantitative analyses for COL I, COL III, VCAM-1, and ICAM-1 expression in the CAVSMCs treated with pEX4-PRDX2 and si-PRDX2 transfection (^*^p < 0.05 and ^**^p < 0.01 vs. pEX4-PRDX2-NC group, ^###^*p* < 0.001 and ^####^*p* < 0.0001 vs. respective si-PRDX2-NC group, ^&^*p* < 0.05, ^&&^*p* < 0.01, and ^&&&^*p* < 0.001 vs. control group). GAPDH was used as an endogenous control. The data were obtained from three independent experiments.

The results of CCK-8 showed that the proliferation of the CAVSMCs was found the lowest in pEX4-PRDX2-CAVSMCs and the highest in siRNA-PRDX2-CAVSMCs at 24 h (Figures 4A,B). The H_2_O_2_ concentration was lower in pEX4-PRDX2-CAVSMCs than in pEX4-PRDX2-NC-CAVSMCs and control. The higher H_2_O_2_ concentration was observed in si-PRDX2-CAVSMCs compared with si-PRDX2-NC-CAVSMCs and control (Figure 4Ca). The H_2_O_2_ assay results indicated that PRDX2 was able to reverse the effects of TNF-α treatment (Figure 4Cb). Meanwhile, Figure 4D showed the consistent trend of ROS expression. As shown in Figure 4E, up-regulating PRDX2 inhibited CAVSMCs migration, whereas down-regulating PRDX2 augmented CAVSMCs migration. These results suggested that PRDX2 played a key role in the inhibition of CAVSMCs generation, H_2_O_2_ production, and migration. The Western blot results indicated that PRDX2 was able to reverse the effects of TNF-α treatment. The expression of COL I, COL III, VCAM-1, and ICAM-1 was decreased in the pEX4-PRDX2-CAVSMCs+TNF-α group compared with the TNF-α group and pEX4-PRDX2-NC-CAVSMCs+TNF-α group (Figure 4F). These results indicated that PRDX2 was a pivotal factor involved in the inhibition of TNF-α-induced progression of AS. Furthermore, the COL I, COL III, VCAM-1, and ICAM-1 expression was decreased in pEX4-PRDX2-CAVSMCs and increased in si-PRDX2-CAVSMCs (Figure 4G). Overall, these data confirmed that PRDX2 protected against the progression of AS through inhibiting the accumulation of collagen, VCAM-1, and ICAM-1, which might be attributed to ROS scavenging.

### PRDX2 Inhibits the Development of AS *via* the MAPK Signaling Pathway

**Figure 5:**
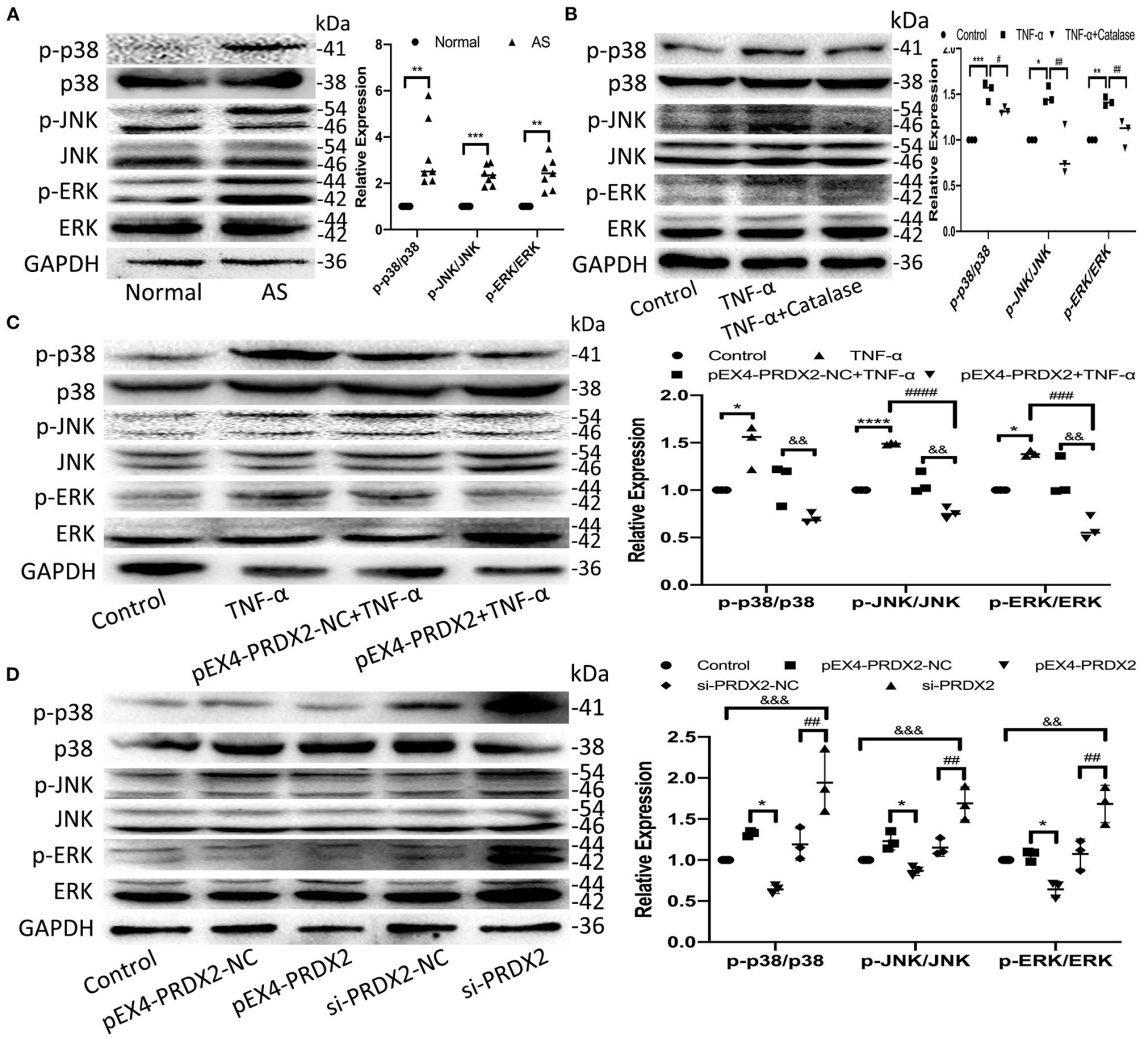
PRDX2 inhibits the development of AS *via* the MAPK signaling pathway. **(A)** The expression of p-p38, p-ERK, and p-JNK was determined by Western blot in human carotid artery specimens (*n* = 7 humans per group, ^**^p < 0.01 and ^***^p < 0.001 vs. normal group). **(B)** The expression of p-p38, p-JNK, and p-ERK was determined by Western blot in the CAVSMCs treated TNF-α and catalase (^*^p < 0.05, ^**^p < 0.01, and ^***^p < 0.001 vs. control group, ^#^*p* < 0.05 and ^##^*p* < 0.01 vs. TNF-α group). **(C)** Western blot analyses for p-p38, p-ERK, and p-JNK in pEX4-PRDX2-CAVSMCs treated with TNF-α (^*^p < 0.05 and ^****^p < 0.0001 vs. control group, ^###^*p* < 0.001 and ^####^*p* < 0.0001 vs. TNF-α group, ^&&^*p* < 0.01 vs. pEX4-PRDX2-NC+TNF-α group). **(D)** Western blot analyses for p-p38, p-JNK, and p-ERK expression in the CAVSMCs treated with pEX4-PRDX2 and si-PRDX2 transfection (^*^p < 0.05 vs. pEX4-PRDX2-NC group, ^##^*p* < 0.01 vs. si-PRDX2-NC group, ^&&^*p* < 0.01 and ^&&&^*p* < 0.001 vs. control group). The data were obtained from three independent experiments.

The high expression of p38, JNK, and ERK phosphorylation was detected in the human carotid AS tissue (Figure 5A). Furthermore, the levels of p-p38, p-JNK, and p-ERK were elevated after TNF-α treatment, which was reversed by catalase treatment (Figure 5B). As shown in Figure 5C, PRDX2 was able to reverse the effects of TNF-α treatment in activating p38, JNK, and ERK (Figure 5C). Furthermore, PRDX2 up-regulation significantly decreased the p-p38, p-JNK, and p-ERK levels, whereas the p-p38, p-JNK, and p-ERK levels were increased when PRDX2 was silenced (Figure 5D). These results indicated that the MAPK signaling pathway was involved in the protection of PRDX2 against AS.

**Figure 6:**
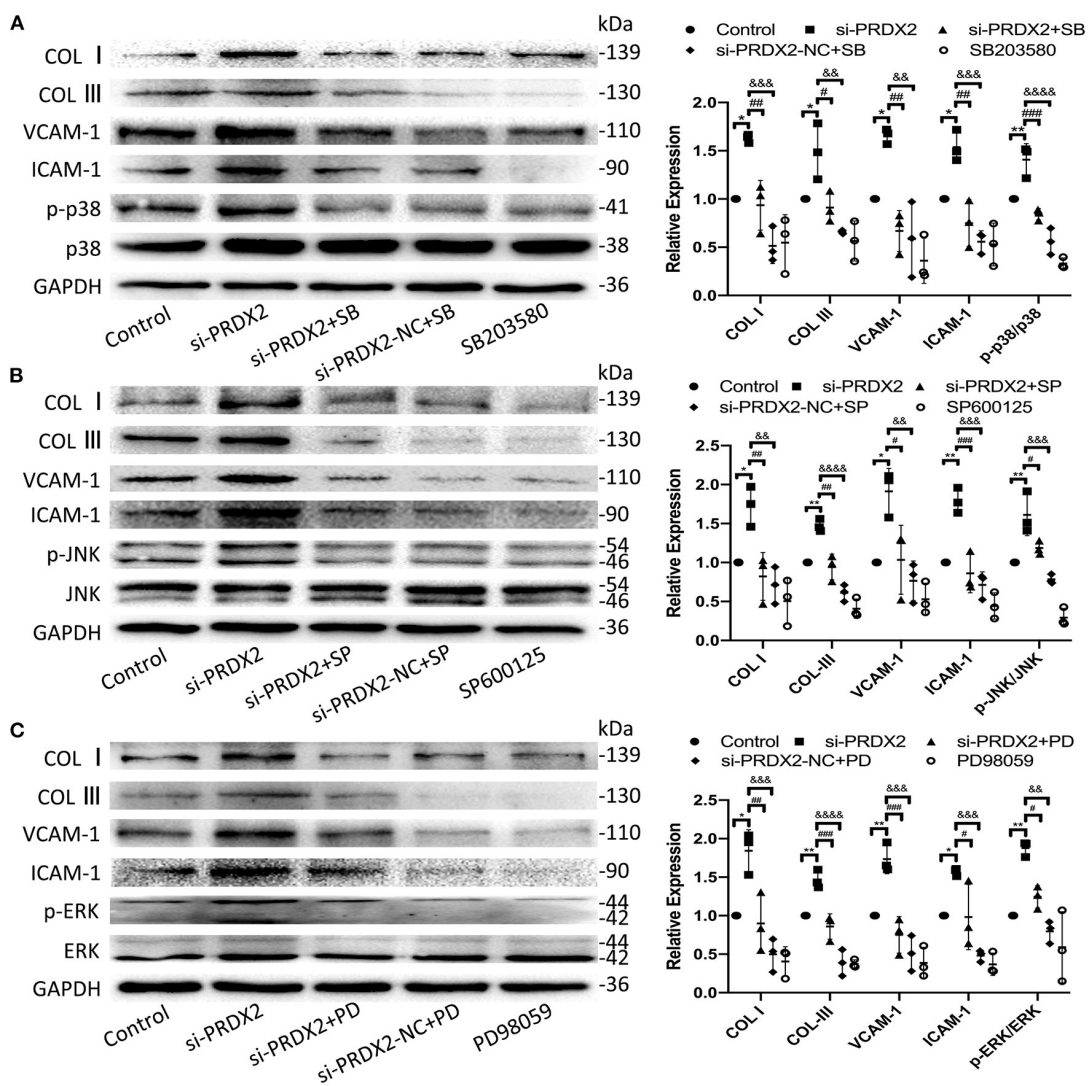
Down-regulation of PRDX2 promotes the potential role of AS by activating the MAPK signaling pathway. **(A)** COL I, COL III, VCAM-1, ICAM-1, and p-p38 protein expression was determined by Western blot in the CAVSMCs treated with SB203580 (^*^p < 0.05 and ^**^p < 0.01 vs. control group, ^#^*p* < 0.05, ^##^*p* < 0.01, and ^###^*p* < 0.001 vs. si-PRDX2+SB group, ^&&^*p* < 0.01, ^&&&^*p* < 0.001, and ^&&&&^*p* < 0.0001 vs. si-PRDX2-NC+SB group). **(B)** COL I, COL III, VCAM-1, ICAM-1, and p-JNK protein expression was determined by Western blot in the CAVSMCs treated with SP600125 (^*^p < 0.05 and ^**^p < 0.01 vs. control group, ^#^*p* < 0.05, ^##^*p* < 0.01, and ^###^*p* < 0.001 si-PRDX2+SP group, ^&&^*p* < 0.01, ^&&&^*p* < 0.001, and ^&&&&^*p* < 0.0001 vs. si-PRDX2-NC+SP group). **(C)** COL I, COL III, VCAM-1, ICAM-1, and p-ERK protein expression was determined by Western blot in the CAVSMCs treated with PD98059 (^*^p < 0.05 and ^**^p < 0.01 vs. control group, ^#^*p* < 0.05, ^##^*p* < 0.01, and ^###^*p* < 0.001 vs. si-PRDX2+PD group, ^&&^*p* < 0.01, ^&&&^*p* < 0.001, and ^&&&&^*p* < 0.0001 vs. si-PRDX2-NC+PD group).

To further confirm the potential mechanisms underlying the protection of PRDX2 against AS, the drug of MAPK signaling pathway inhibitors SB203580, SP600125, and PD98059 was used to inhibit the p38, JNK, and ERK pathways, respectively, in si-PRDX2-CAVSMCs. In the CAVSMCs treated with MAPK inhibitors, the expression of COL I, COL III, VCAM-1, and ICAM-1 protein induced by silencing PRDX2 was significantly blocked in the CAVSMCs treated with MAPK inhibitors (Figures 6A–C). Therefore, these results suggest that the down-regulation of PRDX2 leads to the excessive accumulation of COL I, COL III, VCAM-1, and ICAM-1, which could be the result of the activation of p38, ERK, and JNK signals. Collectively, these results indicate that PRDX2 attenuates COL I, COL III, VCAM-1, and ICAM-1 excessive accumulation, potentially as a result of MAPK signaling pathway inhibition.

## Discussion

There are six subtypes of the PRDXs family, among which PRDX1 and PRDX2 are the most abundant in the PRDXs family. PRDX2 is an antioxidant enzyme that protects the cells by removing ROS (14, 15). ROS, the production of the imbalance of oxidative stress, play critical roles in the regulation of various cell functions and pathological processes, including AS (16, 17). In our study, the protein expression of PRDX2 and the concentration of H_2_O_2_ were markedly increased in the human carotid AS tissue compared with the normal human carotid artery tissue, which suggested that both of them were involved in the progression of AS. It has been extensively discussed in the literature that the TNF-α is involved in AS progression, promoting the development of atherosclerotic lesions (18, 19). We found that the H_2_O_2_ concentration was increased in the CAVSMCs treated with TNF-α compared with the control after 24 h, which was reversed by catalase treatment. In immunofluorescence staining, relatively low expression of ROS in pEX4-PRDX2-CAVSMCs was observed. In contrast, the ROS expression in si-PRDX2-CAVSMCs showed the opposite trend. Meanwhile, PRDX2 successfully suppressed TNF-α-induced ROS in the CAVSMCs. Furthermore, we found that the up-regulation of PRDX2 decreased the concentration of H_2_O_2_ in the CAVSMCs, whereas the silencing of PRDX2 increased the concentration of H_2_O_2_ in the CAVSMCs. Collectively, these indicated that PRDX2 potentially protected against the development of AS *via* scavenging ROS.

It was reported in the literature that the deficiency of PRDX2 accelerated plaque formation *via* endothelial cells and immune cells *in vivo* (10). In the present study, it was observed an increasing of PRDX2 in the human carotid AS tissue, with the main positive location in the intima VSMCs. Much evidence supports the idea that the AS lesion formation is related to VSMCs migration through the internal elastic lamina into intimal areas and proliferation in the plaque (20). The VSMCs, which are not terminally differentiated, maintain remarkable plasticity, and potential of reversible alteration in phenotype even in adult organisms (21, 22). The most significant finding in our study was the increasing expression of PRDX2 in the proliferative VSMCs that showed the unique organ-like arrangement in the intima, which indicated that this VSMCs population underwent abnormal differentiation. It increased the reason to speculate in the intima, the VSMCs suffered the genetic modification, which might be associated with PRDX2. In the present study, the up-regulation of PRDX2 promoted the expression of SM22α, whereas the down-regulation of PRDX2 inhibited the expression of SM22α. These indicate that PRDX2 might potentially inhibit the transformation of the VSMC in the development of AS. Furthermore, the stimulation of VSMCs proliferation and migration with TNF-α was abrogated in the up-regulation of PRDX2. Meanwhile, the down-regulation of PRDX2 increased the VSMCs proliferation and migration, which is the consequence of phenotypic switching (23). Similarly, PRDX2 attenuating the proliferation and migration was observed in hepatocellular carcinoma cells (24). In addition, in our study, it has been demonstrated that the catalase, a scavenger of H_2_O_2_, might reverse the TNF-α-induced proliferation and migration in the CAVSMCs. It contributed to explore the mechanisms underlying PRDX2, ROS scavenger, in preventing AS progression. Taken together, these further confirmed that PRDX2 plays a pivotal role in inhibiting VSMCs phenotypic conversion, proliferation, and migration in the progression of AS *via* scavenging ROS.

Collagen is one of the main components of the ECM in AS plaque (25). Collagens actively participate in arterial wall remodeling during atherogenesis. It is well-studied and documented in the literature that local variations in fibrillar collagen are critical to arterial stiffening and the progression of AS (26). In our study, the increasing and redistribution of COL I and COL III were observed in the human carotid AS tissue. VSMCs are the major source of various factors and ECM that primarily contain COL I and COL III (27, 28). The collagen synthesis capacity of VSMCs is regulated by many factors (29, 30). To further elucidate the potential protective effect of PRDX2 against the development of AS, we detected the important proteins that influence AS progression, including COL I, and COL III in pEX4-PRDX2-CAVSMCs, si-PRDX2-CAVSMCs, and TNF-α with or without catalase treatment. In our study, we found that the expression of COL I and COL III was up-regulated in si-PRDX2-CAVSMCs and TNF-α treatment as expected. This result was further supported by our finding that the over-expression of PRDX2 was able to attenuate the production of COL I and COL III and reverse the effects of TNF-α treatment. Interestingly, TNF-α-induced accumulation of COL I and COL III was suppressed by catalase. These findings further support that PRDX2 inhibits the excessive COL I and COL III *via* the regulation of ROS. Collectively, our results suggested that PRDX2 has an inhibition effect on the production of collagens, which confirmed that PRDX2 protected against the progression of AS *via* regulating the ROS accumulation.

VCAM-1 and ICAM-1 are known to play crucial roles in the homeostasis of blood vessels (31). The VCAM-1 increased in VSMCs with treatment of TNF-α, which play a key role in AS (32). ICAM-1, a member of the immunoglobulin super-family, is widely expressed in various cells under the pathological condition, enhancing the adhesion between cells and vascular endothelium (33–35). The expression of adhesion molecules in lesions can affect the tissue of the cells, promote the production of cytokines and growth factors and the migration of medial VSMCs to the inner membrane, and affect the replication of the cells (36). In the present study, we found that VCAM-1 and ICAM-1 were observed to have higher expression in the human carotid artery AS tissue than in the normal human carotid artery tissue. Both the up-regulation of PRDX2 and catalase successfully reversed the TNF-α-induced VCAM-1 and ICAM-1 in the CAVSMCs. The present study demonstrated that the expression of VCAM-1 and ICAM-1 was enhanced in si-PRDX2-CAVSMCs and decreased in pEX4-PRDX2-CAVSMCs. These results further strengthened the evidence that PRDX2 exerted an inhibitor effect on the production of VCAM-1 and ICAM-1 that promoted the development of AS through suppressing ROS.

The MAPK, a member of the mitogen-activated serine/threonine kinase family, closely associates with AS progression in the regulation of VSMCs proliferation and function (37, 38). We found that the up-regulation of PRDX2 reversed the TNF-α-induced phosphorylation of p38, JNK, and ERK. Meanwhile, similar results were gained in catalase treatment, which inhibited the TNF-α-induced activation of p38, JNK, and ERK in the CAVSMCs. In particular, the activation of p38, JNK, and ERK was significantly increased after PRDX2 was down-regulated, suggesting that PRDX2 could block the MAPK signaling pathway in the CAVSMCs. It is reported that the VSMCs in atherosclerotic lesions expressed VCAM-1 and ICAM-1 that would be stimulated by the MAPK signaling pathway (39, 40). SB203580, SP600125, and PD98059 pathway inhibitors have been shown to inhibit the MAPK signaling pathway (41). The present study showed that PRDX2 silencing promoted the expression levels of MAPK signaling and their downstream molecules, whereas si-PRDX2-induced COL I, COL III, VCAM-1, and ICAM-1 syntheses were reduced by treatment with SB203580, SP600125, and PD98059, respectively. Taken together, PRDX2 inhibited the expression of COL I, COL III, VCAM-1, and ICAM-1 in the CAVSMCs *via* regulating the MAPK signaling pathway.

In conclusion, our results suggest a protective role of PRDX2, as a scavenger of ROS, in AS progression through inhibiting the VSMC phenotype alteration and function *via* MAPK signaling pathway.

## Study Limitations

First, in the present study, 14 samples were obtained from autopsy within 48 h after death. In the future, more human samples studies are needed. Meanwhile, more details, such as the expression of PRDX2 and associated molecules in blood plasma, deserve a discussion. Second, our data provide evidence for the next PRDX2 animal model research to discuss the protective function of PRDX2 against AS in the VSMCs. Thus, the *in vivo* experiments are needed.

## Data Availability Statement

The original contributions presented in the study are included in the article/Supplementary Material, further inquiries can be directed to the corresponding author/s.

## Ethics Statement

The studies involving human participants were reviewed and approved by the Ethics Committee of Dalian Medical University. Written informed consent to participate in this study was provided by the participants' legal guardian/next of kin.

## Author Contributions

JL performed the analyses and wrote the draft paper. CW did the statistical analyses and wrote the statistical part of the paper. WW, LL, and QZ were responsible for sample handling, storage, and analysis. BW, SW, and LH contributed to the idea and analysis of the literature. CG performed the morphological analysis. XY and LS conceived the study and contributed to the writing of the manuscript. All authors critically reviewed the manuscript and contributed to its content.

## Conflict of Interest

The authors declare that the research was conducted in the absence of any commercial or financial relationships that could be construed as a potential conflict of interest.
