# Supplementary figures and images for: PRDX2 Protects Against Atherosclerosis by Regulating the Phenotype and Function of the Vascular Smooth Muscle Cell
Source: Front Cardiovasc Med. 2021 Mar 11;8:624796. doi: 10.3389/fcvm.2021.624796 (PMC8006347; doi:10.3389/fcvm.2021.624796)

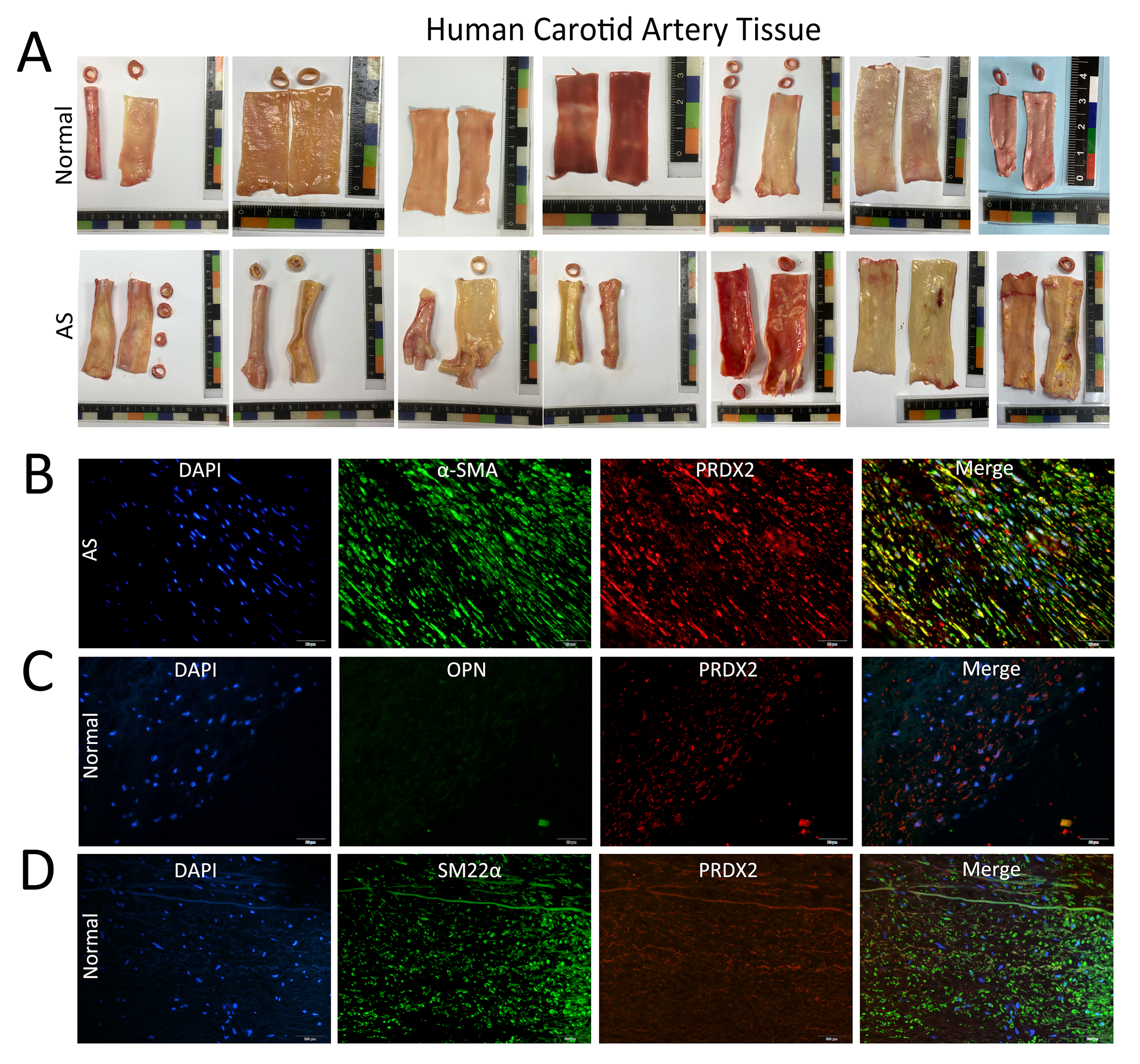

Supplement: Supplementary file 1 [file Image_1.TIF]
