# Supplementary material for: PRDX2 Protects Against Atherosclerosis by Regulating the Phenotype and Function of the Vascular Smooth Muscle Cell
Source: Front Cardiovasc Med. 2021 Mar 11;8:624796. doi: 10.3389/fcvm.2021.624796 (PMC8006347; doi:10.3389/fcvm.2021.624796)
Supplement: Supplementary file 2 [file Data_Sheet_1.PDF]

Selected Human Case Information

Order	Group	Age	Sex	Cause of Death	History of past illness	AS
1	Control	52	M	Aconitine poisoning	no	no
2	Control	27	M	The skull repair operation failed, leading to multifunctional organ failure and death	no	no
3	Control	38	M	Cellulitis at the base of the mouth develops into pus; blood inhalation lung asphyxiation and death	no	no
4	Control	49	M	Sudden cardiac death under the action of external forces	no	no
5	Control	39	M	Alcohol poisoning causes respiratory failure and death	no	no
6	Control	27	F	Commiting traffic offences. Death due to respiratory and circulatory failure.	no	no
7	Control	30	M	Facial soft tissue cellulitis; facial edema; asphyxia	no	no
8	AS	73	M	Acute myocardial infarction causing sudden cardiac death	no	yes
9	AS	30	M	Coronary heart disease; acute myocardial infarction; sudden cardiac death	coronary heart disease	yes
10	AS	66	M	Sudden cardiac death was caused by allergic reaction to infusion and bleeding in the left anterior descending artery plaque	hypertensive disease	yes
11	AS	62	M	Atherosclerosis of coronary artery;Acute infarction; sudden cardiac death;	coronary heart disease	yes
12	AS	70	M	Surgical incision infection; intestinal obstruction	no	yes
13	AS	69	M	Cellulitis of the floor of the mouth; Throat spasmodic	no	yes
14	AS	62	M	Large area myocardial infarction; myocardial ischemia; myocardial anoxia ;	coronary heart disease	yes
